# A novel metabolite-interacting protein (MIP)-based molecular subtyping construction and validation of IGFBP3 as a MIP-related oncogene in colorectal cancer

**DOI:** 10.1016/j.gendis.2024.101272

**Published:** 2024-03-22

**Authors:** Simeng Bao, Yongmin Li, Yu Liang, Jianxun Dai, Huolin Huang, Bin Ma

**Affiliations:** a Central Laboratory, Cancer Hospital of China Medical University, Cancer Hospital of Dalian University of Technology, Liaoning Cancer Hospital & Institute, Shenyang, Liaoning 110042, China; b Department of Colorectal Surgery, Cancer Hospital of China Medical University, Cancer Hospital of Dalian University of Technology, Liaoning Cancer Hospital & Institute, Shenyang, Liaoning 110042, China; c School of Optoelectronic Engineering and Instrumentation Science, Dalian University of Technology, Dalian, Liaoning 116024, China; d The Liaoning Provincial Key Laboratory of Interdisciplinary Research on Gastrointestinal Tumor Combining Medicine with Engineering, Shenyang, Liaoning 110042, China

For prognosis and therapeutic response, colorectal cancer (CRC) patients are highly variable across stages, correlated to high inter-tumor heterogeneity at the molecular level.^1^ Therefore, molecular subtyping needs to be determined for stratifying patients into distinct prognostic subgroups according to tumor biology.^2^ Metabolic reprogramming is an important cancer hallmark and confers a few cancer phenotypes.^3^ Metabolic landscape, comprising metabolites, proteins, and their interactions remains high variability across pan-cancer or even the same cancer type with distinct conditions.^4^^,^^5^ Thus, comprehending the transcriptional changes of metabolite-interacting proteins (MIPs) may provide valuable insights into promising therapeutic targets. In the present study, CRC patients were categorized into two subtypes, namely C1 and C2 based on prognostic MIPs, and each subtype owned a distinct clinical outcome. Meanwhile, an MIP-relevant risk score was generated and could accurately predict survival outcomes and personalized therapy for CRC based on bioinformatics mining. Additionally, a MIP-relevant gene, insulin-like growth factor-binding protein 3 (IGFBP3) was overexpressed in CRC tissues, and deficiency of *IGFBP3* could facilitate the accumulation of intracellular reactive oxygen species and inhibit mitophagy in CRC cells. In summary, the current study proposed robust MIP-based molecular subtyping and relevant risk scores for guiding therapy selection and prognosis prediction of CRC patients, which might facilitate comprehension and clinical application for CRC metabolism heterogeneity.

Firstly, we integrated transcriptional expression profiling and clinical data of colon and rectal cancers from TCGA, and adjusted batch effects (Fig. S1A). In total, 4293 MIPs were collected, and following the cutoffs of |fold change| ≥2 and adjusted *P* < 0.01, 434 MIPs exhibited significant differential expression in CRC versus normal tissues, with 257 down-regulations and 177 up-regulations (Fig. S1B). Among them, 56 MIPs were significantly linked to CRC prognosis, which were adopted for consensus clustering analysis. In accordance with the cumulative distribution function curve, the optimal number of clusters was 2 across TCGA-CRC, namely C1 and C2 (Fig. S1C–F). The prognostic MIPs were differently expressed between the two MIP subtypes (Fig. S1G). Principal component analysis proved the accuracy in subtype assignment (Fig. S1H). Overall survival outcomes differed between subtypes, with poorer overall survival for C2 (Fig. S1I). Next, clinicopathological traits were compared, more advanced histological stage, N and M stage, and more dead cases were observed in C2 versus C1 (Fig. S1J).

We then screened MIP-relevant genes through weighted gene co-expression network analysis. This analysis incorporated the genes with variance within the top 5000 (Fig. S2A). The optimal power value was identified as 9 based on scale independence >0.9 and relatively high mean connectivity (Fig. S2B). Totally, eight co-expression modules were constructed (Fig. S2C). Magenta and green modules exhibited the strongest Pearson correlation with MIP subtypes (Fig. S2D), which were regarded as key modules. The genes were extracted from key modules, which were regarded as MIP-relevant genes. Additionally, we observed notable relationships of module membership in the magenta and green modules with gene significance for MIP subtypes (Fig. S2E, F). The 703 genes in the magenta and green modules were regarded as MIP-relevant genes. Notably, the genes in the magenta module were linked to CRC tumorigenic pathways (Fig. S2G, H), while those in the green module were mainly correlated to immunity (Fig. S2I, J).

Further definition and external verification of a MIP-relevant gene signature for CRC was carried out. Table S1 listed the 27 prognostic MIP-relevant genes in TCGA-CRC, which were input into the least absolute shrinkage and selection operator analysis (Fig. S3A, B). Then, a MIP-relevant gene signature was generated, following the formula: risk score = 0.0662321391578679 ∗ ALDH1A3 expression + 0.0961678919845657 ∗ OLFM2 expression + 0.0824843559063323 ∗ SLC2A3 expression + 0.130463406568411 ∗ GPC1 expression + 0.00922543892229505 ∗ IGFBP3 expression + 0.0804414140611728 ∗ GRP expression + −0.111871932923389 ∗ F2RL2 expression + 0.00585574851488039 ∗ MEGF6 expression + −0.343287749363947 ∗ WNT5A expression + 0.203137325063818 ∗ TIMP1 expression (Fig. S3C). With the median RiskScore, we classified TCGA-CRC as low- and high-risk groups (Fig. S3D), and principal component analysis demonstrated the accuracy of grouping assignment (Fig. S3E). Among TCGA-CRC cases, high-risk scores correlated to poorer overall survival outcomes (Fig. S3F), with the area under the curves at 1-, 3- and 5-year overall survival >0.6 (Fig. S3G), indicating the predictive potential in CRC prognosis. The generalizability of this MIP-relevant risk score was proven in the GSE39582 cohort (Fig. S3H, I).

We then evaluated the MIP-relevant risk score in predicting the recurrence and progression of CRC. Our analysis showed that high-risk score was correlated to worse disease-free survival (Fig. S4A, B), disease-specific survival (Fig. S4C, D), and progression-free survival (Fig. S4E, F) across TCGA-CRC, proving that the MIP-relevant risk score might enable to predict recurrence and progression of CRC. In Figure S4G, more advanced histological stage, TNM stage, and more dead cases were investigated in the high-risk group. We further explored the relationship of the MIP-relevant risk score with clinical response to immune checkpoint blockade. Further analyses found that the MIP-relevant risk score positively correlated to most steps within the cancer-immunity cycle (Fig. S5A). In addition, risk score exhibited positive associations with stromal activation, with negative associations with DNA damage repair (Fig. S5B). Next, we validated the predictive efficacy of risk score in immune checkpoint blockade response in the IMvigor210 cohort. Low-risk cases had relatively higher proportions of partial and complete responses to anti-PD-L1 therapy (Fig. S5C, D) and displayed better overall survival (Fig. S5E). Among three immune phenotypes that were classified by the spatial distribution of CD8^+^ T cells, desert tumors possessed lower risk scores than excluded or inflamed tumors (Fig. S5F). Compared with IC0 (immune cells with the lowest PD-L1), a higher risk score was observed in IC2 (immune cells with the highest PD-L1) (Fig. S5G). Additionally, TC2+ tumors with the highest PD-L1 displayed higher risk scores than TC0 or TC1 tumors with the lowest or modest PD-L1 (Fig. S5H). Overall, the MIP-relevant risk score correlated to the inflamed tumor microenvironment.

To determine the expression level of MIP-related risk model genes, we tested the expression of 10 genes in our collected CRC tissues and found that IGFBP3 exhibited the most significant differential expression among these genes (Fig. 1A, B). The expression of IGFBP3 was significantly increased in matching liver metastatic samples compared with primary CRC and adjacent noncancerous tissue by immunohistochemistry in the cohort of patients (Fig. 1C, D). Additionally, subsequent analyses revealed that higher expression of IGFBP3 was associated with worse clinicopathologic characteristics in CRC patients (Table S2). Furthermore, Kaplan–Meier curve analysis demonstrated that high expression of IGFBP3 was associated with lower overall survival (Fig. 1E). Moreover, multivariate Cox regression analysis indicated that IGFBP3 was an independent prognostic factor (Fig. 1F). To further evaluate the oncogenic role of IGFBP3 *in vitro*, as shown in Figure 1G–K and Figure S7A–C, we found that the cellular viability, proliferation, and migration were significantly decreased in *IGFBP3*-KD cells. Further RNA sequencing analysis showed that expression levels of mitophagy-related genes were obviously reduced in *IGFBP3*-KD cells (Fig. S6). The mitochondria of *IGFBP3*-KD cells had shorter fragments than Scramble cells and the density of mitochondria decreased in *IGFBP3*-KD cells (Fig. 1L, M). Meanwhile, the analysis of flow cytometry and fluorescence intensity showed that *IGFBP3* deficiency significantly increased the generation of reactive oxygen species in CRC cells (Fig. 1N–S; Fig. S7D–F). Additionally, the levels of LC3-I and P62 were increased in *IGFBP3*-KD CRC cells (Fig. 1T, U). More importantly, the levels of mitophagy proteins PINK1, PARKIN, and BNIP3L were decreased in *IGFBP3*-KD cells (Fig. 1T, U; Fig. S7G).

**Figure 1:**
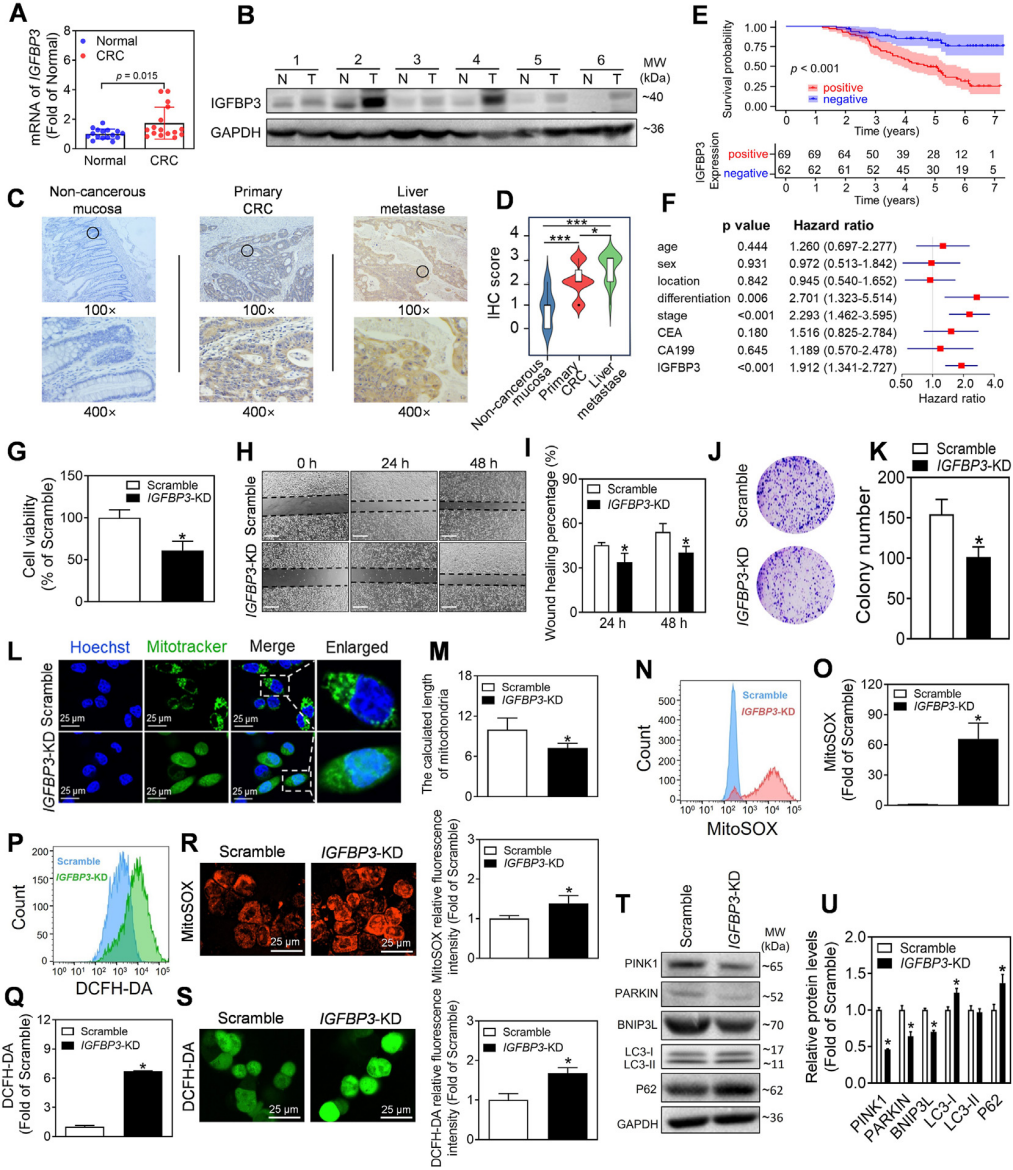
IGFBP3 plays an oncogenic role in colorectal cancer (CRC) by regulating mitophagy. **(A)** Quantitative reverse transcription PCR analysis of *IGFBP3* in CRC and adjacent normal tissues. *n* = 16. ∗*P* < 0.05 *vs*. adjacent normal tissues. **(B)** Immunoblot analysis of IGFBP3 in CRC and adjacent normal tissues. Representative images were shown. *n* = 6. T: CRC tissues; N: adjacent normal tissues. **(C)** Representative immunohistochemistry images of IGFBP3 in noncancerous mucosa, paired primary CRC and liver metastases. **(D)** Quantification analysis of (C). ∗*P* < 0.05, ∗∗∗*P* < 0.001. **(E)** Kaplan–Meier plots of CRC specimens with negative and positive IGFBP3 expression. **(F)** Multivariate Cox regression of IGFBP3 expression and clinical parameters in CRC. **(G)** Cell viability of *IGFBP3*-KD HCT116 cells was detected by CCK8 assay following 24-h culture. *n* = 3. ∗*P* < 0.05 *vs*. Scramble cells. **(H, I)** Representative images and quantification analysis of wound healing assay in *IGFBP3* deficient HCT116 cells. The images were taken at the indicated time points. *n* = 3. ∗*P* < 0.05 *vs* Scramble cells with the same time point. Scale bar = 500 μm. **(J, K)** Representative images and quantification analysis for a colony formation assay in *IGFBP3*-deficient HCT116 cells. *n* = 3. ∗*P* < 0.05 *vs*. Scramble cells. **(L, M)***IGFBP3*-KD cells were stained with Mito-Tracker Green to evaluate the mitochondrial morphology, and the mitochondrial length was analyzed quantitatively with image J. *n* = 3. ∗*P* < 0.05 *vs*. Scramble cells. Blue: Hoechst staining; Green: Mito-Tracker staining. Scale bar = 25 μm. **(N, O)***IGFBP3*-KD HCT116 cells were stained with MitoSOX and analyzed by flow cytometry. MitoSOX-positive cells were quantitatively analyzed with FlowJo. *n* = 3. ∗*P* < 0.05 *vs*. Scramble cells. **(P, Q)***IGFBP3*-KD HCT116 cells were stained with DCFH-DA and analyzed by flow cytometry. DCFH-DA-positive cells were quantitatively analyzed with FlowJo. *n* = 3. ∗*P* < 0.05 *vs*. Scramble cells. **(R)** Representative images and quantification analysis of mitochondrial reactive oxygen species (ROS) in *IGFBP3*-deficient HCT116 cells. *n* = 3. ∗*P* < 0.05 *vs*. Scramble cells. Red: MitoSOX staining. Scale bar = 25 μm. Fluorescence intensity was quantitatively analyzed with image J. **(S)** Representative images and quantification analysis of intracellular ROS in *IGFBP3*-KD cells. *n* = 3. ∗*P* < 0.05 *vs*. Scramble cells. Green: DCFH-DA staining. Scale bar = 25 μm. **(T, U)** Immunoblotting and quantification analysis of mitophagy-related proteins (PINK1, PARKIN, and BNIP3L) and autophagy-related proteins (LC3-I, LC3-II, and P62) in *IGFBP3*-KD HCT116 cells. *n* = 3. ∗*P* < 0.05 *vs*. Scramble cells.

Altogether, we propose robust MIP-based molecular subtyping and relevant RiskScore for guiding therapy selection and prognosis prediction of CRC, thus facilitating comprehension and clinical application for CRC metabolism heterogeneity.

## Ethics declaration

The research involving human participants was approved by the human ethics committees of the Liaoning Cancer Hospital (No. 20210804 GP). The patients/participants provided their written informed consent to participate in the study.

## Author contributions

B.M. and S.B. conceived this research. B.M. and Y.L. performed the bioinformatic analysis and visualization. S.B. designed and performed the *in vitro* experiments with contributions from B.M., Y.L., J.D., and H.H. S.B. and B.M. analyzed data and wrote the manuscript. All authors revised and approved the final manuscript.

## Funding

This work was supported by grants from the Cultivation Program of National Science Foundation of Liaoning Cancer Hospital (China) (No. 2021-ZLLH-03), Beijing Xisike Clinical Oncology Research Foundation (China) (No. Y-QL202101-0039), Medical and Industrial Cross Joint Fund of Dalian University of Technology-Liaoning Cancer Hospital (China) (No. LD2023027), The National Natural Science Foundation of China (No. 82303131), and the Liaoning Province Science and Technology Plan Joint Program (Applied Basic Research Project) (China) (No. 2023JH2/101700150).

## Data availability

The datasets generated and analyzed in this study are all from public databases, and the main raw dataset comes from the TCGA database (https://portal.gdc.cancer.gov/) and GSE39582 dataset (https://www.ncbi.nlm.nih.gov/geo/query/acc.cgi?acc=GSE39582).

## Conflict of interests

The authors declared no conflict of interests.
