# Supplementary material for: A novel metabolite-interacting protein (MIP)-based molecular subtyping construction and validation of IGFBP3 as a MIP-related oncogene in colorectal cancer
Source: Genes Dis. 2024 Mar 22;12(2):101272. doi: 10.1016/j.gendis.2024.101272 (PMC11585693; doi:10.1016/j.gendis.2024.101272)
Supplement: Multimedia component 1 [file mmc1.docx]

**Supplementary Material**

**Supplementary materials and methods**

**Collection of CRC cohorts**

RNA sequencing with transcripts per million (TPM) format covering 51 normal and 647 CRC tissues of colon (COAD) and rectal cancers (READ) was retrieved from TCGA (https://portal.gdc.cancer.gov/) as the training cohort. Among them, 600 cases possessed complete clinicopathological information. Batch effect was adjusted through sva package.^1^ Somatic mutation as well as copy number variations (CNVs) with “Masked Copy Number Segment” format were gathered from TCGA. The transcriptional expression profiling and prognostic information (n=579) of the GSE39582 dataset were utilized as the validation cohort from the GEO repository (http://www.ncbi.nlm.nih.gov/geo).^2^

**Analysis of differential expression**

Totally, 4293 MIPs were extracted from four databases comprising Kyoto Encyclopedia of Genes and Genomes (KEGG; http://www.kegg.jp/),^3^ Reactome (http://www.reactome.org),^4^ Metabolic Atlas (https://www.metabolicatlas.org/)^5^ together with BRENDA (www.brenda-enzymes.org).^6^The expression of MIPs in normal and CRC tissues at the transcriptional level was measured through limma package.^7^ Adjusted p values for multiple testing were computed with Benjamini–Hochberg method. The cutoffs were set as |fold change (FC)|≥2 and adjusted *p* < 0.01.

**Consensus clustering**

Prognostic MIPs were selected across TCGA-CRC through univariate cox regression approach, which were input into consensus clustering utilizing ConsensusClusterPlus package.^8^ The optimal number of clusters was chosen in accordance with cumulative distribution function (CDF) curves. Principal component analysis (PCA) was utilized for proving the subtype assignment based on the transcriptional expression profiling.

**Functional annotation**

The gene sets of “H.all.v7.2.symbols.gmt” hallmarks and known biological processes were gathered from the MSigDB (http://software.broadinstitute.org/gsea/msigdb)^9^ and previously published literature. GSVA package was adopted for quantifying the relative enrichment levels.^10^ Biological processes and KEGG pathways of MIP-relevant genes were annotated utilizing clusterProfiler package.^11^ False discovery rate (FDR)<0.05 was regarded as significant enrichment.

**Immunotherapeutic response**

Tumor immune dysfunction and exclusion (TIDE) computational approach was adopted for inferring ICB response.^12^ Immunophenoscore (IPS) of 597 CRC patients was gathered from The Cancer Immunome Database (TCIA; https://www.tcia.at/home) that offers integrated immunogenomic analysis of next-generation sequencing data across pan-cancer from TCGA and others. Transcriptional expression profiling and matched clinical information of IMvigor210 immunotherapeutic cohort were gathered from http://research-pub.gene.com/imvigor210corebiologies. After normalization, the counts were converted to TPM format.

**Weighted Gene Co-expression Network Analysis (WGCNA)**

WGCNA can transform expression profiling into co-expression gene modules and explore the relationships between modules and phenotype features.^13^ Firstly, the genes with variance within the top 5000 were chosen for WGCNA. Scale independence together with mean connectivity were measured under a gradient of soft threshold power values (ranging from 1 to 20). The optimal power value was identified when scale independence was >0.9 and mean connectivity was relatively high. Afterwards, the adjacency matrix was converted to topological overlap matrix (TOM). In accordance with TOM-based dissimilarity, genes were classified as distinct co-expression modules. The minimal module size was set as 30, with cut height as 0.25. Module eigengene (ME) was regarded as a main principal component of a given co-expression module. The modules with the strongest Pearson correlation with MIP phenotypes were considered as key modules. The genes were extracted from key modules, a total of 703 genes were obtained from the two most relevant modules (green and magenta), and then univariate and [multivariate Cox analyses](https://fanyi.baidu.com/mtpe-individual/multimodal?channel=pcLeftFileButton) were performed separately. In order to further clarify the oncogenic effect of genes in CRC, we intersected significantly up-regulated genes and genes with prognostic significance in CRC, and finally we obtained 27 genes regarding as MIP-relevant genes.

**Signature construction**

The prognostic significance of MIP-relevant genes was evaluated utilizing univariate cox regression approach. Those with *p* < 0.05 were entered into least absolute shrinkage and selection operator (LASSO) regression for dimension reduction. The optimal tuning parameter (λ) value was chosen through ten-time cross-validation utilizing the minimum criteria. The formula of MIP-relevant gene signature was computed via standardizing the transcriptional level of selected genes together with matched coefficients, as follows: riskscore = $\sum_{i=1}^{n} Gi*\beta i$, where n denotes the number of prognostic MIP-relevant genes; Gi denotes the expression level of gene I; βi denotes the coefficient of gene i. The median riskscore as a cut-off value classified CRC as high- and low-risk groups. PCA was adopted for verifying the two groups using the transcriptional expression profiling. Time-dependent receiver operating characteristic (ROC) curve was conducted for assessing the prediction accuracy. The riskscore was externally verified in the GSE39582 cohort.

**Human CRC samples**

Clinical data of patients with CRC and tissue samples (CRC samples, paired adjacent noncancerous tissues and matching liver metastasis specimens) were obtained from Liaoning Cancer Hospital and Institute (Shenyang, China). The CRC samples were confirmed to have a histological diagnosis of CRC. The study was approved by the institutional ethics committee and the collection of all tissue samples was carried out in accordance with the informed consent policy.

**Immunohistochemistry**

The tissue sections were incubated with IGFBP3 antibody (sc-374365; RRID: AB_10988386; 1:500) purchased from Santa Cruz, Inc. (Santa Cruz, CA). Based on the average staining intensity and the percentage of positively stained cells, the quantification of IGFBP3 expression was accomplished through the Image J software (NIH Image, Bethesda, MD). The IGFBP3 IHC staining was evaluated using a scoring system ranging from "−" to "+", and from "++" to "+++", indicating negative and positive results, respectively.

**Cell culture and reagents**

HCT116 (CL-0096) and SW620 cells (CL-0225B) were purchased from Procell Life Science & Technology Co. Ltd. (Wuhan, China). The culture medium for HCT116 cells was McCoy’s 5A containing 10% fetal bovine serum (FBS) and 1% penicillin–streptomycin. The culture medium for SW620 cells was high glucose DMEM containing 10% fetal bovine serum (FBS) and 1% penicillin–streptomycin.

HCT116 and SW620 cells were cultured in a 37 °C 95% air and 5% CO_2_ incubator (Thermo Scientific, US). McCoy’s 5A and high glucose DMEM were purchased from Procell Life Science & Technology Co. Ltd. (Wuhan, China). FBS, penicillin–streptomycin and phosphate buffered saline were purchased from Biological Industries (BioInd, Israel).

**Lentiviral-based short-hairpin RNA (shRNA) transduction**

The shRNA vector targeting *IGFBP3* and control vector were transfected into 293T cells together with 2 packaging vectors. A lentivirus containing shRNA against *IGFBP3* or a scrambled shRNA control was transduced into HCT116 and SW620 cells. Puromycin was added to the cells after transduction at a concentration of 3.0 μg/mL.

**Cell viability assay**

Scramble and *IGFBP3*-KD cells were seeded in 96-well plates (5×10^4^ cells per well) and allowed to adhere for 24 hours at 37 °C. The viability of the cells was analyzed using the cell counting kit-8 (Glpbio) according to the manufacturer’s instruction.

**Wound healing assay**

Scramble and *IGFBP3*-KD cells were seeded in 6-well plates (1×10^6^ cells per well) overnight. The cell monolayers were scratched using a sterile pipette tip. The culture medium was replaced and the images of the wound healing were taken after 0, 24 and 48 h of incubation by a phase-contrast microscope. The distance between the edges of the wound was measured by Image J software.

**Colony formation assay**

Scramble and *IGFBP3*-KD cells were seeded in 6-well plates (10^3^ cells per well). The cells were fixed with 4% paraformaldehyde and stained with crystal violet solution after 10 days of culture. The number of colonies was counted and the images were taken using a phase-contrast microscope.

**Measurement of ROS**

Intracellular ROS levels were detected by MitoSOX Red (M36008, Invitrogen) and DCFH-DA (S0033, Beyotime) fluorescent probe. The cells were washed by using PBS and subsequently incubated with serum-free DMEM containing 5 μM DCFH-DA and 2.5 μg/mL MitoSOX respectively. The cells were cultured in a 37 °C for 30 min. The generation of intracellular ROS was measured by flow cytometry. The images were acquired by using Yokogawa cell voyager CQ1.

**Mito-Tracker Green staining**

Mito-Tracker Green was used to detect the mitochondrial morphology and the methods referred to the instructions of Mito-Tracker Green kit (KGMP007, KeyGEN BioTECH, China). The images were acquired by using Yokogawa cell voyager CQ1.

**Western blot analysis**

Whole-cell extract preparation and Western blotting were performed as detailed previously.^14^ Antibody for IGFBP3 (sc-374365; RRID: AB_10988386; 1:1000) was purchased from Santa Cruz, Inc. (Santa Cruz, CA). Antibodies against PINK1 (#23274-1-AP; RRID: AB_2879244; 1:1000), PARKIN (#14060-1-AP; RRID: AB_2878005; 1:1000), P62 (#18420-1-AP; RRID: AB_10694431; 1:1000), LC3 (#14600-1-AP; RRID: AB_2137737; 1:1000) and BNIP3L (#12986-1-AP; RRID: AB_2877901; 1:1000) were purchased from Proteintech Group, Inc. (Wuhan, China). Antibody for GAPDH (TA-08; RRID: AB_2747414; 1:1000) was purchased from Beijing Zhongshan Jinqiao Biological Technology Co. (Beijing, China). The protein expression levels of each sample were normalized by GAPDH levels, and density of each band was determined using the Image J software.

**Quantitative RT-PCR**

Total RNA was extracted from CRC tissue samples and HCT116 cells by using TRIzol reagent (Life Technologies, Carlsbad, CA) and quantified by Nanodrop 2000 (Thermo, Wilmington, DE). PrimeScript™ RT reagent Kit with gDNA Eraser (TaKaRa, Dalian, China) was used to reverse-transcribe total RNA into cDNA. SYBR mix (TaKaRa, Dalian, China) was utilized for conducting Realtime PCR and the fluorescence detection was performed using QuantStudio 6 Flex (ABI, Foster City, CA). The primer sequences were as follows: *IGFBP3* (forward 5’-AGAGCACAGATACCCAGAACT-3’ and reverse 5’-GGTGATTCAGTGTGTCTTCCATT-3’), *GAPDH* (forward 5’-GGAGCGAGATCCCTCCAAAA-3’ and reverse 5’-GGCTGTTGTCATACTTCTCATGG-3’).

**RNA-seq data analysis**

Total RNA from Scramble and *IGFBP3*-KD HCT116 cells was extracted with TRIzol reagent (Life Technologies, Carlsbad, CA), and then the RNA-sequencing was performed by SEQHEALTH Company (Wuhan, China).

**Statistical analysis**

All computational and statistical analyses were implemented with R language (https://www.r‐project.org/). Unpaired Student’s t‐test was applied for comparing two groups with normally distributed variables, while Wilcoxon test was utilized for non‐normally distributed variables. Overall survival (OS), disease-free survival (DFS), disease-specific survival (DSS) together with progression-free survival (PFS) analyses were conducted with survival package, with log‐rank test for determining survival difference. Spearman correlation test was adopted for inferring the association between two variables that were not linearly correlated. *p* < 0.05 indicated statistical difference. Statistical analysis of molecular biology experiments was performed using GraphPad Prism 5 (GraphPad Software, San Diego, CA), with a *p* < 0.05 considered as significant. All the data were expressed as the mean ± standard deviation (SD). Statistical differences were determined by a Student's *t*-test or by two-way ANOVA followed by Bonferroni post hoc test.

**Supplementary references**

1 Leek JT, Johnson WE, Parker HS, et al. The sva package for removing batch effects and other unwanted variation in high-throughput experiments. *Bioinformatics*. 2012;28(6):882-883.

2 Marisa L, de Reyniès A, Duval A, et al. Gene expression classification of colon cancer into molecular subtypes: characterization, validation, and prognostic value. *PLoS Med*. 2013;10(5):e1001453.

3 Kanehisa M, Sato Y, Kawashima M, et al. KEGG as a reference resource for gene and protein annotation. *Nucleic Acids Res*. 2016;44(D1):D457-462.

4 Croft D, O'Kelly G, Wu G, et al. Reactome: a database of reactions, pathways and biological processes. *Nucleic Acids Res*. 2011;39(Database issue):D691-697.

5 Robinson JL, Kocabaş P, Wang H, et al. An atlas of human metabolism. *Sci Signal*. 2020;13(624).

6 Placzek S, Schomburg I, Chang A, et al. BRENDA in 2017: new perspectives and new tools in BRENDA. *Nucleic Acids Res*. 2017;45(D1):D380-d388.

7 Ritchie ME, Phipson B, Wu D, et al. limma powers differential expression analyses for RNA-sequencing and microarray studies. *Nucleic Acids Res*. 2015;43(7):e47.

8 Wilkerson MD, Hayes DN. ConsensusClusterPlus: a class discovery tool with confidence assessments and item tracking. *Bioinformatics*. 2010;26(12):1572-1573.

9 Liberzon A, Birger C, Thorvaldsdóttir H, et al. The Molecular Signatures Database (MSigDB) hallmark gene set collection. *Cell Syst*. 2015;1(6):417-425.

10 Hänzelmann S, Castelo R, Guinney J. GSVA: gene set variation analysis for microarray and RNA-seq data. *BMC Bioinformatics*. 2013;14:7.

11 Yu G, Wang LG, Han Y, et al. clusterProfiler: an R package for comparing biological themes among gene clusters. *Omics*. 2012;16(5):284-287.

12 Jiang P, Gu S, Pan D, et al. Signatures of T cell dysfunction and exclusion predict cancer immunotherapy response. *Nat Med*. 2018;24(10):1550-1558.

13 Langfelder P, Horvath S. WGCNA: an R package for weighted correlation network analysis. *BMC Bioinformatics*. 2008;9:559.

14 Bao S, Zheng H, Chen C, et al. Nfe2l1 deficiency mitigates streptozotocin-induced pancreatic β-cell destruction and development of diabetes in male mice. *Food Chem Toxicol*. 2021;158:112633.

**
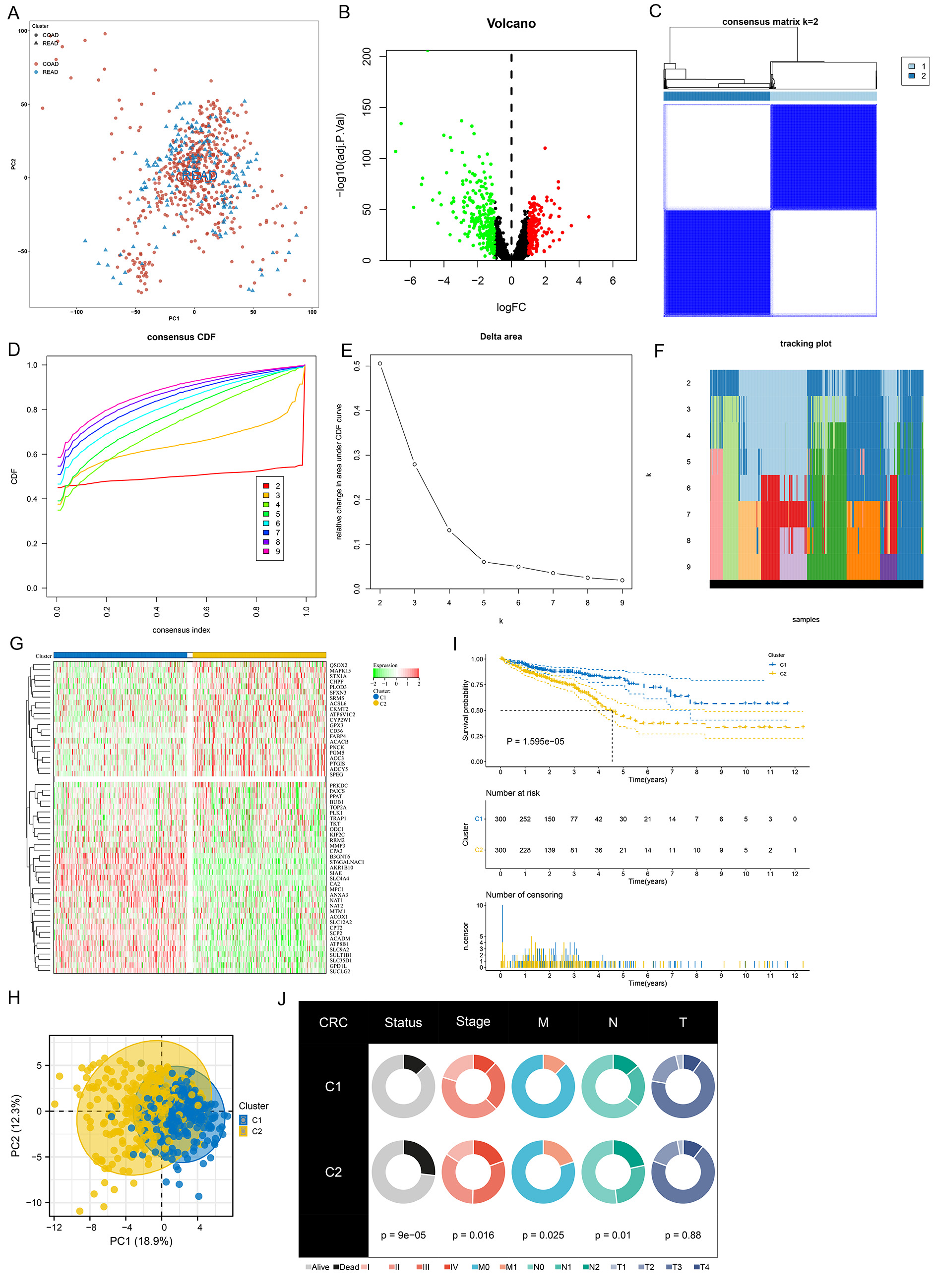
Supplementary figures**

**Fig. S1: Generation of two MIP subtypes with distinct OS and clinicopathological traits across TCGA-CRC.** (A) PCA for the integrated RNA-seq data of colon (COAD) and rectal cancers (READ) from TCGA. (B) Volcano diagram visualizing the MIPs with differential expression in CRC versus normal tissues. Green, down-regulation; red, up-regulation. (C) Consensus matrix heatmap, (D) CDF curve, (E) delta area curve, (F) tracking plot on the basis of expression profiling of prognostic and differentially expressed MIPs across TCGA-CRC. (G) Transcriptional levels of prognostic and differentially expressed MIPs in the two MIP subtypes. (H) PCA for proving the subtype assignment in accordance with the transcriptional expression profiling. (I) K-M curve of OS between subtypes. (J) Distribution of clinicopathological traits across the two MIP subtypes.


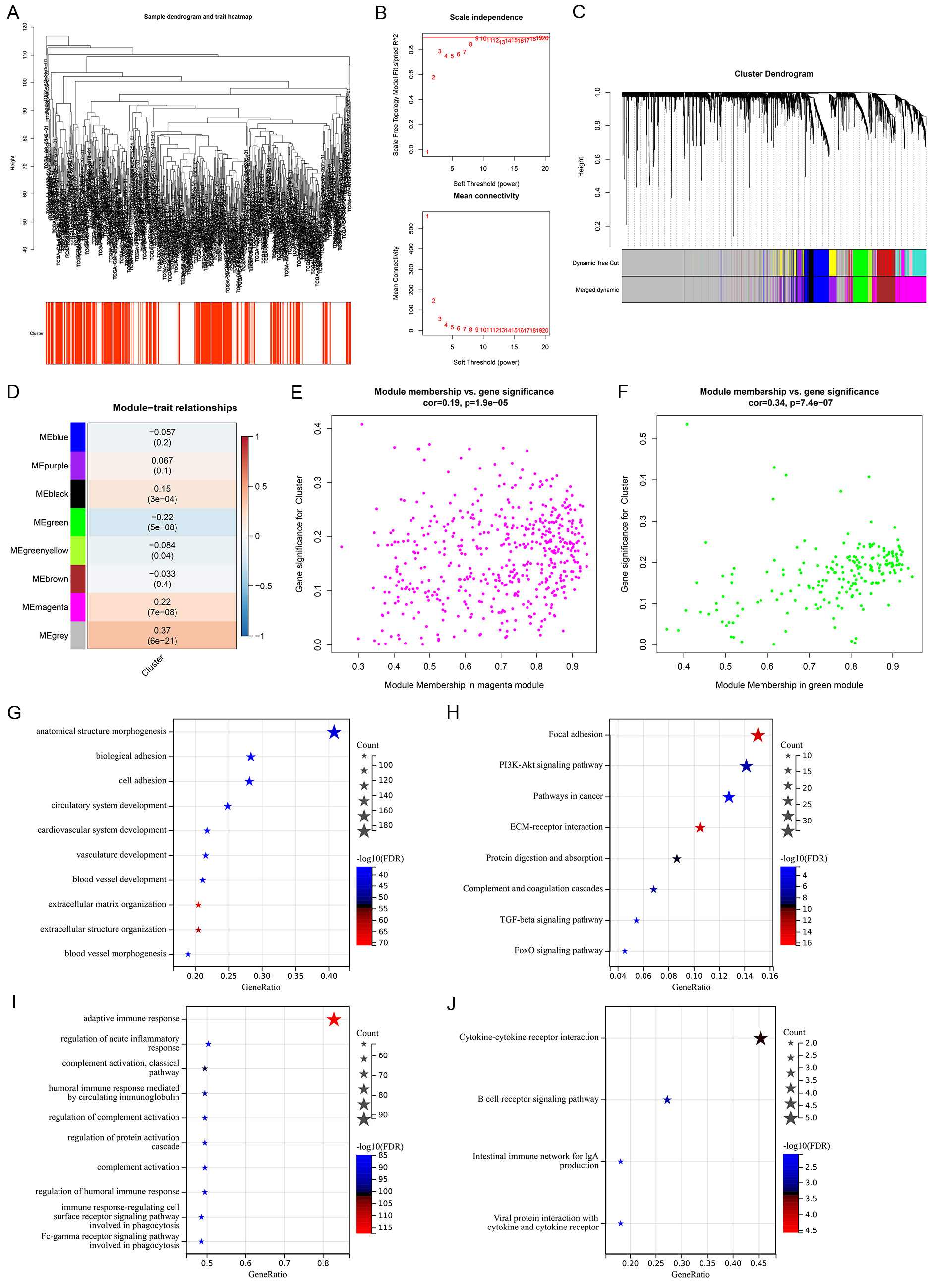


**Fig. S2: Screening MIP-relevant genes through WGNCA.** (A) Heatmap of sample dendrogram and MIP classification. (B) Scale independence together with mean connectivity of a gradient of soft-thresholding powers from 1 to 20. (C) Cluster dendrogram on the basis of a dissimilarity measure. (D) Relationships of MEs and MIP subtypes. (E, F) Scatter plots for relationships of module membership in magenta and green modules with gene significance for MIP subtypes. (G, H) Biological processes and KEGG pathways of genes in the magenta module. (I, J) Biological processes and KEGG pathways of genes in the green module.


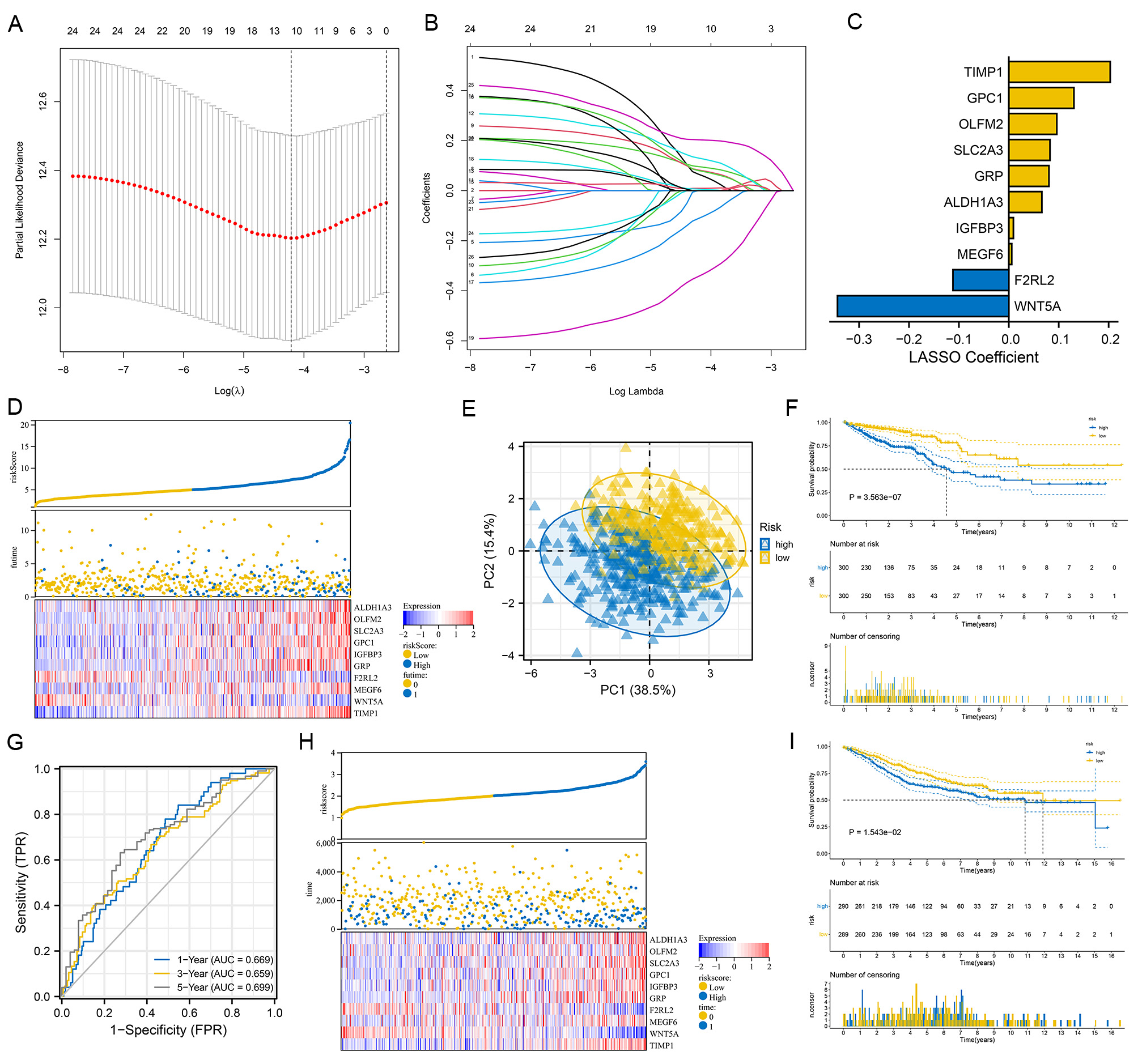


**Fig. S3: Definition of a MIP-relevant gene signature in TCGA-CRC and external verification.** (A) Partial likelihood deviation curve. (B) LASSO coefficients under a gradient of lambda values. (C) LASSO coefficients of genes in the MIP-relevant gene signature. (D) Distribution of RiskScore, survival status together with transcriptional levels of MIP-relevant genes. (E) PCA for proving the grouping assignment on the basis of the transcriptional expression profiling. (F) K-M curve of OS between low- and high-risk cases. (G) ROC curve of the MIP-relevant riskscore at 1-, 3- and 5-year OS. (H, I) External verification of K-M curve of OS as well as ROC curve in the GSE39582 cohort.


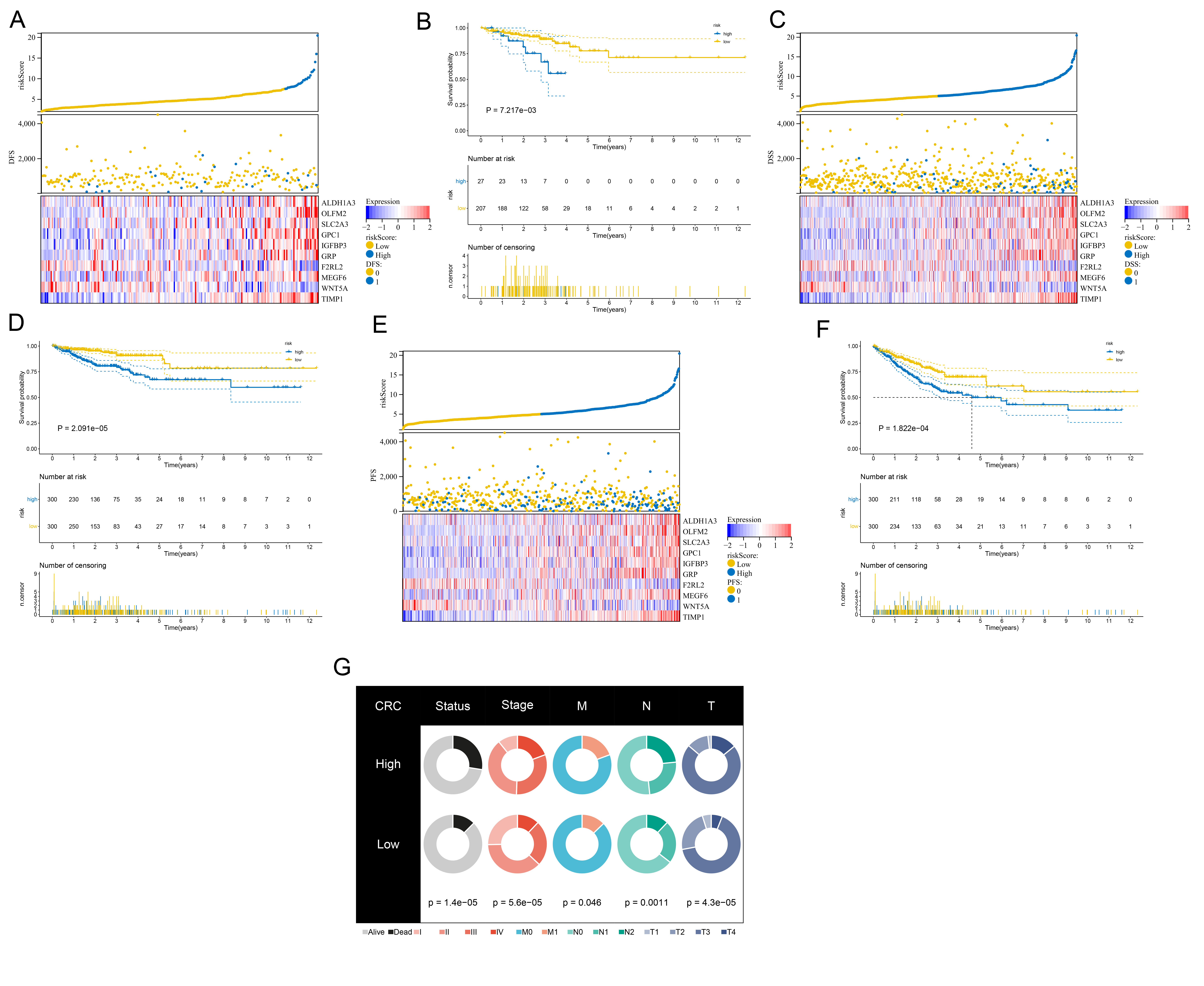


**Fig. S4: Evaluation of the MIP-relevant RiskScore in predicting recurrence and progression and establishment of a prognostic nomogram in TCGA-CRC.** (A) Distribution of RiskScore, recurrence status, and transcription levels of MIP-relevant genes. (B) K-M curve of DFS between low- and high-risk cases. (C) Distribution of RiskScore, DSS status together with transcription levels of MIP-relevant genes. (D) K-M curve of DSS between low- and high-risk cases. (E) Distribution of RiskScore, progression status together with transcription levels of MIP-relevant genes. (F) K-M curve of PFS between low- and high-risk cases. (G) Distribution of clinicopathological traits of low- and high-risk groups.


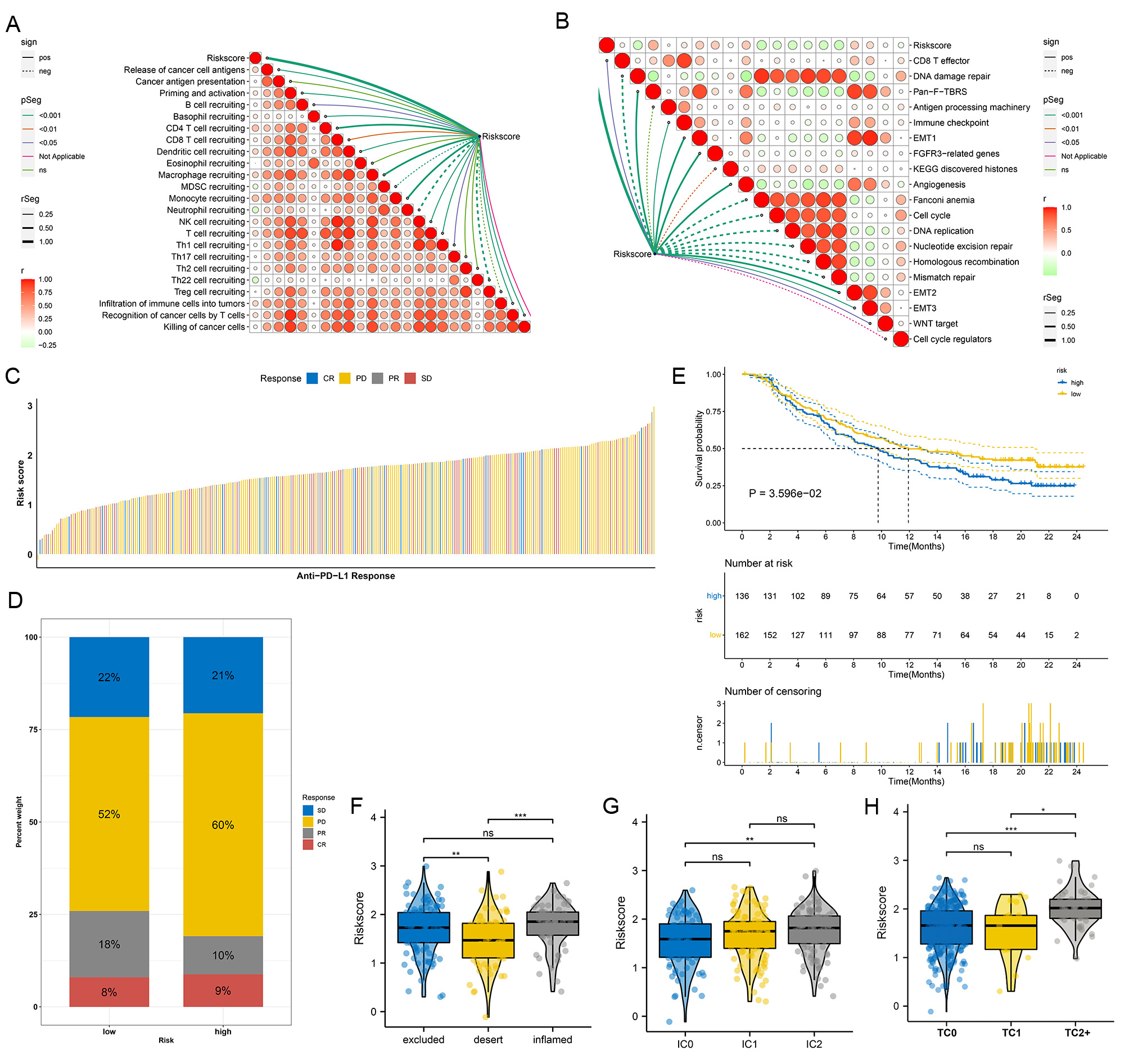


**Fig. S5: Relationship of the MIP-relevant riskscore with clinical response to immunotherapy.** (A, B) Associations of riskscore with activity of cancer-immunity cycle and known biological processes. Small to large circles indicate weak to strong correlations. Green, negative correlation; red, positive correlation. (C) Distribution of riskscore across patients who differently responded to anti-PD-L1 therapy in the IMvigor210 cohort. (D) Proportions of distinct overall responses including stable disease (SD), progressive disease (PD), partial response (PR), and complete response (CR) in low- and high-risk patients after anti-PD-L1 treatment in the IMvigor210 cohort. (E) K-M curve of OS between low- and high-risk patients in the IMvigor210 cohort. (F-H) Comparison of riskscore among different immune phenotypes, IC and TC levels. **p* < 0.05; ***p* < 0.01; ****p* < 0.001; ns: *p* > 0.05.


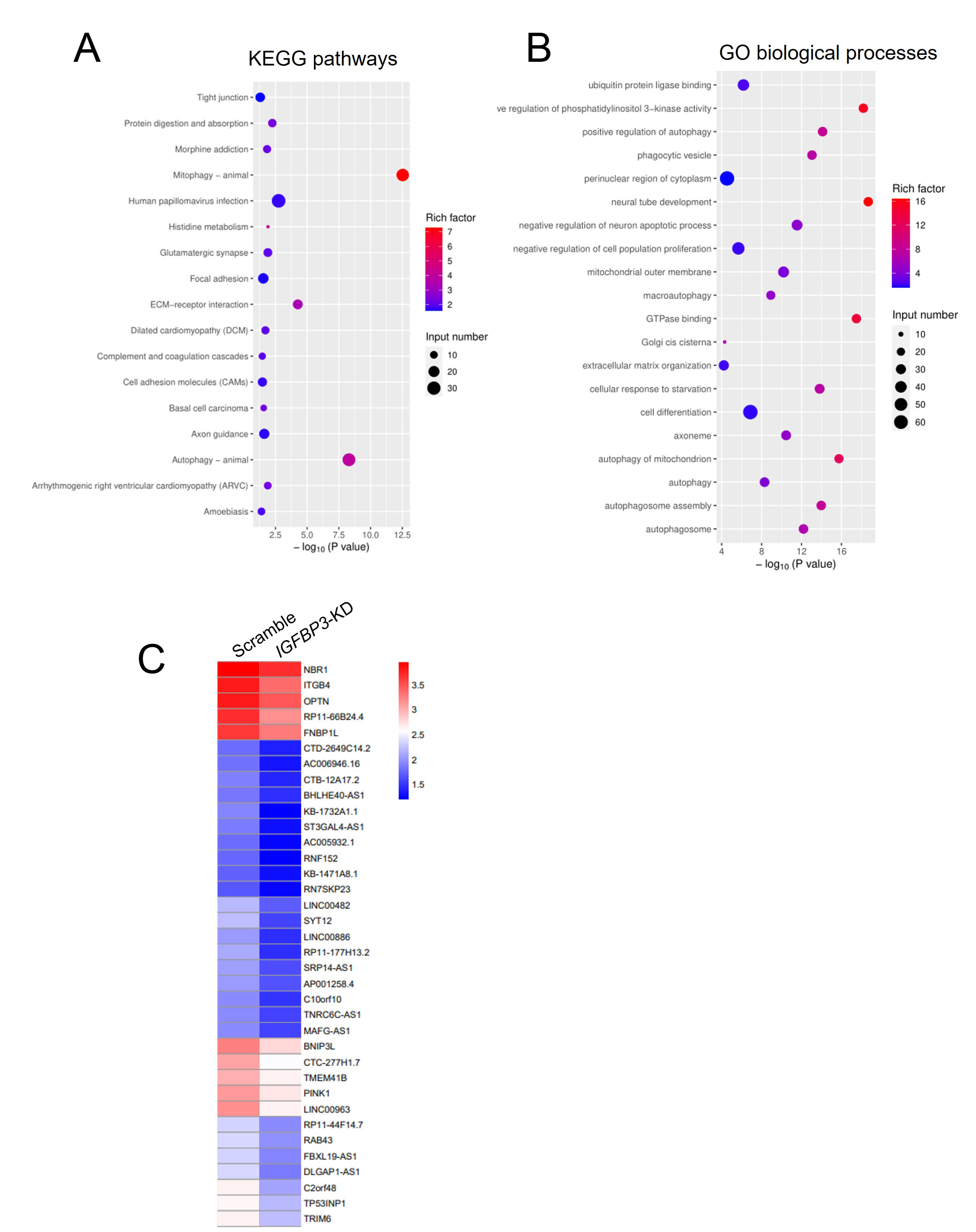


**Fig. S6: The RNA-seq analysis of *IGFBP3*-deficient HCT116 cells.** (A) KEGG pathway enrichment analysis of the transcriptome data of *IGFBP3*-KD HCT116 cells. (B) GO biological processes. (C) The heatmap of mitophagy and autophagy-related genes from RNA-seq data.


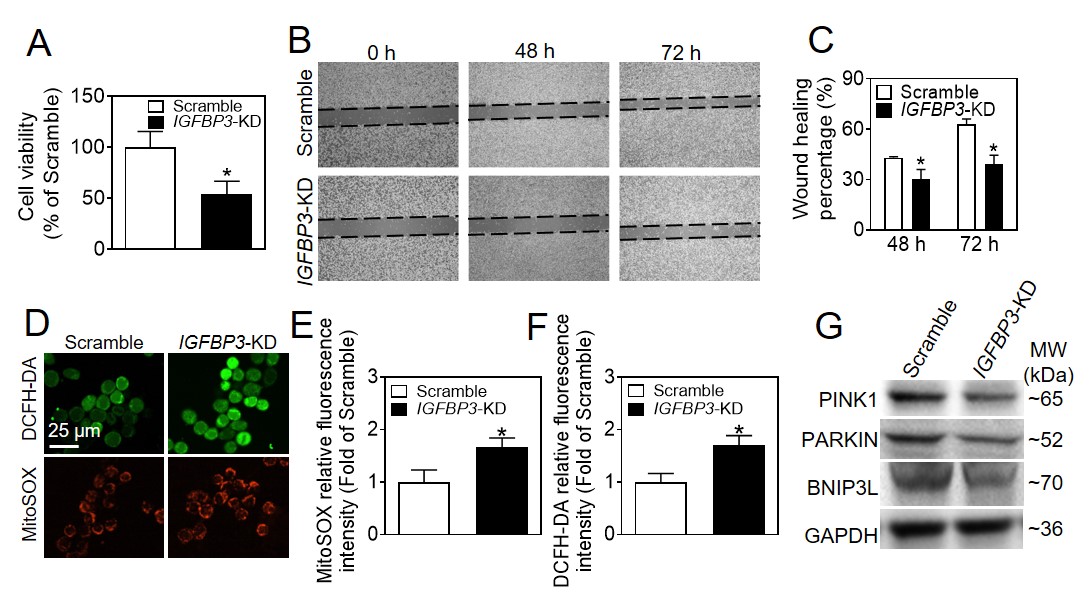


**Fig. S7: *IGFBP3* deficiency suppressed cell viability, migration and promoted intracellular ROS generation in SW620 cells.** (A) Cell viability of *IGFBP3*-KD SW620 cells was detected by CCK8 assay following 24 h culture. n = 3 **p* < 0.05 vs Scramble cells. (B, C) Representative images and quantification analysis of wound healing assay in *IGFBP3* deficient SW620 cells. The images were taken at the indicated time points. n = 3. **p* < 0.05 vs Scramble cells with the same time point. (D-F) Representative images and quantification analysis of intracellular ROS in *IGFBP3*-deficient SW620 cells. n = 3. **p* < 0.05 vs Scramble cells. Green: DCFH-DA staining. Red: MitoSOX staining. Scale bar = 25 μm. Fluorescence intensity was quantitatively analyzed with image J. (G) Immunoblotting of mitophagy-related proteins (PINK1, PARKIN and BNIP3L) in *IGFBP3*-KD SW620 cells. n = 3.

**Supplementary tables**

**Table S1. Twenty-seven prognostic MIP-relevant genes in CRC.**

| ID | HR | 95%lower | 95%upper | P-value |
| --- | --- | --- | --- | --- |
| ALDH1A3 | 1.322902 | 1.033778 | 1.692888 | 0.026149 |
| HOMER3 | 1.515913 | 1.193851 | 1.924855 | 0.00064 |
| PRRX2 | 1.241544 | 1.021252 | 1.509356 | 0.029933 |
| OLFM2 | 1.312022 | 1.096273 | 1.570231 | 0.003049 |
| ISM1 | 1.241956 | 1.001935 | 1.539477 | 0.047973 |
| MXRA8 | 1.184626 | 1.000853 | 1.402143 | 0.048853 |
| GJA4 | 1.285217 | 1.01278 | 1.630939 | 0.038976 |
| PLA1A | 1.298021 | 1.055168 | 1.596767 | 0.013584 |
| SLC2A3 | 1.240483 | 1.045241 | 1.472196 | 0.013649 |
| FHL3 | 1.311591 | 1.040494 | 1.653322 | 0.021678 |
| EVA1B | 1.263647 | 1.029075 | 1.551688 | 0.025514 |
| GPC1 | 1.372968 | 1.122217 | 1.679747 | 0.002066 |
| COL18A1 | 1.207299 | 1.00235 | 1.454154 | 0.047178 |
| RGCC | 1.259613 | 1.041794 | 1.522974 | 0.017189 |
| RCN3 | 1.259813 | 1.043265 | 1.521308 | 0.01639 |
| IGFBP3 | 1.302484 | 1.08508 | 1.563446 | 0.004564 |
| GRP | 1.282845 | 1.063983 | 1.546726 | 0.00906 |
| F2RL2 | 0.734716 | 0.57396 | 0.940497 | 0.014409 |
| MEGF6 | 1.310751 | 1.075609 | 1.597298 | 0.007307 |
| WNT5A | 0.712684 | 0.564562 | 0.899667 | 0.00438 |
| CDR2L | 1.264055 | 1.006807 | 1.587033 | 0.04355 |
| FSTL3 | 1.337645 | 1.127459 | 1.587016 | 0.000852 |
| NOTCH3 | 1.255163 | 1.028007 | 1.532512 | 0.025672 |
| CHPF | 1.373841 | 1.088855 | 1.733416 | 0.007415 |
| COMP | 1.148429 | 1.038242 | 1.27031 | 0.007162 |
| TIMP1 | 1.524987 | 1.230902 | 1.889334 | 0.000113 |
| BGN | 1.161078 | 1.017288 | 1.325193 | 0.026824 |

| **Table S2. IGFBP3 expression and associations with clinicopathologic characteristics in CRC patients.** | | | | | |
| --- | --- | --- | --- | --- | --- |
|  | IGFBP3 expression | | | | |
| Variable | No. | Negative | Positive | χ^2^ | *p*-value |
| **Age(years)** |  |  |  | 0.317 | 0.573 |
| <60 | 60 | 30 | 30 |  |  |
| ≥60 | 71 | 32 | 39 |  |  |
| **Gender** |  |  |  | 5.378 | 0.020* |
| Male | 71 | 27 | 44 |  |  |
| Female | 60 | 35 | 25 |  |  |
| **Location** |  |  |  | 0.405 | 0.525 |
| colon | 63 | 28 | 35 |  |  |
| rectum | 68 | 34 | 34 |  |  |
| **Differentiation** |  |  |  | 0.567 | 0.452 |
| poor | 18 | 10 | 8 |  |  |
| well | 113 | 52 | 61 |  |  |
| **pT stage** |  |  |  | 2.132 | 0.144 |
| T1-T2 | 21 | 13 | 8 |  |  |
| T3-T4 | 110 | 49 | 61 |  |  |
| **pN stage** |  |  |  | 26.306 | <0.001*** |
| N0 | 60 | 43 | 17 |  |  |
| N1-N2 | 71 | 19 | 52 |  |  |
| **pM stage** |  |  |  | 6.419 | 0.011* |
| M0 | 109 | 57 | 52 |  |  |
| M1 | 22 | 5 | 17 |  |  |
| **AJCC stage** |  |  |  | 12.740 | <0.001*** |
| I | 17 | 12 | 5 |  |  |
| II | 36 | 28 | 8 |  |  |
| III | 56 | 17 | 39 |  |  |
| IV | 22 | 5 | 17 |  |  |
| **CEA** |  |  |  | 5.590 | 0.018* |
| ≤5.0(Negative) | 79 | 44 | 35 |  |  |
| >5.0(Positive) | 52 | 18 | 34 |  |  |
| **CA199** |  |  |  | 2.516 | 0.113 |
| ≤37.0(Negative) | 114 | 57 | 57 |  |  |
| >37.0(Positive) | 17 | 5 | 12 |  |  |
| **Total** | 131 | 62 | 69 |  |  |
| Significance level as indicated: *, *p* < 0.05; **, *p* < 0.01; ***, *p* < 0.001. | | | | | |
